# Warburg and Beyond: The Power of Mitochondrial Metabolism to Collaborate or Replace Fermentative Glycolysis in Cancer

**DOI:** 10.3390/cancers12051119

**Published:** 2020-04-30

**Authors:** Shamir Cassim, Milica Vučetić, Maša Ždralević, Jacques Pouyssegur

**Affiliations:** 1 Department of Medical Biology, Centre Scientifique de Monaco, CSM, 98000 Monaco, Monaco; milica@centrescientifique.mc; 2 Centre A. Lacassagne, University Côte d’Azur, IRCAN, CNRS, 06189 Nice, France; masa.zdralevic@gmail.com

**Keywords:** tumor, metabolism, Warburg effect, oxidative phosphorylation (OXPHOS), mitochondria, Krebs cycle, therapy

## Abstract

A defining hallmark of tumor phenotypes is uncontrolled cell proliferation, while fermentative glycolysis has long been considered as one of the major metabolic pathways that allows energy production and provides intermediates for the anabolic growth of cancer cells. Although such a vision has been crucial for the development of clinical imaging modalities, it has become now evident that in contrast to prior beliefs, mitochondria play a key role in tumorigenesis. Recent findings demonstrated that a full genetic disruption of the Warburg effect of aggressive cancers does not suppress but instead reduces tumor growth. Tumor growth then relies exclusively on functional mitochondria. Besides having fundamental bioenergetic functions, mitochondrial metabolism indeed provides appropriate building blocks for tumor anabolism, controls redox balance, and coordinates cell death. Hence, mitochondria represent promising targets for the development of novel anti-cancer agents. Here, after revisiting the long-standing Warburg effect from a historic and dynamic perspective, we review the role of mitochondria in cancer with particular attention to the cancer cell-intrinsic/extrinsic mechanisms through which mitochondria influence all steps of tumorigenesis, and briefly discuss the therapeutic potential of targeting mitochondrial metabolism for cancer therapy.

## 1. Introduction

These past decades have been marked by a growing number of studies dealing with cancer and have led to a better overall understanding of this disease, in particular the fact that (1) cancer is not a disease that could only originate from genetic and epigenetic modifications [1,2,3,4], and (2) tumor cells do not exclusively depend on aerobic glycolysis to satisfy their bioenergetic and anabolic demands [5,6,7,8]. Indeed, mitochondria have now been recognized as important mediators of cancer behavior in all steps of tumorigenesis [9,10]. These long-standing misconceptions actually stem from the “self/non-self” discrimination—which suggests that the immune system can only recognize foreign entities [11,12]—and the so-called “Warburg effect”, referring to Otto Warburg, who described in 1927 an increased ability of tumors to uptake glucose [6,13,14,15]. Nevertheless, these correct, but incomplete assumptions led to a wave of investigations that have revolutionized modern medicine, including the rationale for the development of the [^18^F]-fluorodeoxyglucose-positron emission tomography (FDG-PET) clinical imaging tool in the detection of tumor and metastatic foci [15,16].

As will be detailed hereafter, Warburg’s seminal observation that tumor cells sustain aerobic glycolysis, which corresponds to the fermentation of glucose into lactate when oxygen is present (in contrast to the complete oxidation of glucose by mitochondria), gave prominence to the role of mitochondria in cancer [14]. While the “Warburg effect” is an undeniable characteristic displayed by a majority of tumor cells, Warburg himself suggested that the capacity of neoplastic cells to sustain enhanced glycolytic rates would stem from primary mitochondrial defects [17], thus obscuring the role of mitochondria in cancer for almost 80 years. However, in the mid-1990s, a renewed interest in the relevance of mitochondria in cancer has attributed several pleiotropic roles during tumorigenesis [9,10]. For example, mitochondrial outer membrane permeabilization (MOMP) has been revealed as a critical step in the execution of regulated cell death (RCD) [18,19] and has led to the identification that most cancer cells exhibit an increased resistance to RCD [1,3]. Considerable efforts to identify and develop molecules that would efficiently target these mitochondrial control points have thus been made as a strategy for chemo/radio-sensitization [20,21,22]. From a metabolic perspective, mitochondria attracted renewed attention as it became clear that they can favor cancer reprogramming in imparting substantial metabolic flexibility, thereby allowing tumor cell growth and survival under fluctuating microenvironmental conditions. For example, mitochondrial uncoupling—which is the abrogation of ATP generation in response to the mitochondrial membrane’s potential—has been shown to favor the Warburg effect in tumor cells (for further details on mitochondrial uncoupling and its function in cancer cell metabolism, please refer to [23]). In the same vein, some mitochondrial metabolites have been shown to be sufficient to drive oncogenesis [24], and some mitochondrial metabolic pathways could adapt to serve bioenergetic or anabolic functions, such as during nutrient restriction, low levels of oxygen tension (hypoxia), and cancer treatments [25,26,27,28,29]. Hence, targeting mitochondrial metabolism remains a major but very challenging approach for the establishment of promising cancer therapeutics [30,31,32].

However, one of the biggest challenging issues with targeting mitochondria as a relevant approach to efficiently eradicate tumor cells (or at least make them vulnerable after treatment exposure) is that all differentiated tissues are dependent on oxidative phosphorylation (OXPHOS) to survive [33,34,35]. This would then lead to the establishment of refined therapeutic strategies, whereby tumor cells would be selectively targeted, while non-tumor/normal differentiated cells would be spared from—or unresponsive to—the treatment-derived effects. Here, after revisiting the Warburg effect from a historic/dynamic perspective, and seeing whether or not it is dispensable for cancer, we will discuss the role of mitochondria in cancer by detailing the mechanisms through which they can impact on tumorigenicity, and we will briefly highlight the therapeutic opportunities of targeting mitochondrial metabolism as an anticancer strategy.

## 2. The Warburg Effect: From Myth to Reality

### 2.1. Historical Perspectives

Otto Warburg and colleagues noticed, in the 1920s, that tumors were able to uptake massive amounts of glucose in comparison with normal surrounding tissues. Furthermore, it has been observed that glucose fermentation could occur in these tumors in order to generate lactate even under oxygen non-limiting conditions, thus the term “aerobic glycolysis” [36,37]. However, it was also noticed that respiration alone could preserve tumor cell survival, and it was thus suggested that to proficiently eradicate cancer cells by siphoning them of their energy, both glucose and oxygen had to be removed [13]. In 1925, Cori and Cori extended Warburg’s work and revealed the in situ lactic acid levels displayed by mouse and rat tumors to be very much lower than those observed in the in vitro experiments of Warburg [13,37,38]. Notably, Cori and Cori proved that the blood drawn that emanates from a tumor had considerably reduced glucose and increased lactic acid than the blood passing through the non-tumoral tissues, and finally concluded that this initial excess of lactic acid generation displayed by tumors was washed out by the blood flow through the tissue [38]. In 1929, the English biochemist Herbert Crabtree studied the glycolytic activity of different tumor types and noticed some heterogeneity. While confirming Warburg’s seminal findings, Crabtree further revealed a heterogeneity in the magnitude of respiration exhibited by tumors, with many tumors showing an important capacity to sustain respiration [39]. Hence, Crabtree suggested that not only do cancer cells display enhanced aerobic glycolysis, but that significant differences were also observed during fermentation according to the environmental or genetic variations. 

Warburg later proposed, in 1956, that “the respiration of all cancer cells is damaged”, thereby promoting the incorrect assumption that impaired respiration was the sine qua non that causes the increased fermentation of glucose in tumors, even though observations from his own laboratory and those of others indicated otherwise [14]. The observations of Chance and Weinhouse indeed controverted Warburg’s hypothesis of mitochondrial defects in tumors [17,40], as rat hepatoma cells were evidenced to have active mitochondria and respiratory capacity [41]. However, Warburg used to say that “science progresses not because scientists change their minds, but because scientists attached to erroneous views die, and are replaced” [42]. Warburg actually assumed that respiration must be damaged in cancers because high levels of O_2_ were unable to abrogate the lactic acid production by tumor cells [43]. Nonetheless, in 1962, Warburg attempted to justify the conclusions he had drawn and admitted that the description he made based on insufficient (rather than damaged) respiration had led to “fruitless controversy”. In the early 1970s, Efraim Racker termed this phenomenon the “Warburg effect” and also pinpointed prior findings demonstrating that tumors were able to sustain respiration, and developed his own ideas about the Warburg effect origins: ranging from intracellular pH disparities to ATPase activity alterations [44,45]. Later on, Jeffrey Flier, Racker and Morris Birnbaum confirmed that aerobic glycolysis was a well-regulated process controlled by growth factor signaling [46,47,48,49]. However, at that time, the discovery of oncogenes led to the assumption that aberrant regulation of growth factor signaling was an initiating event in tumorigenesis, shedding new light on Warburg’s hypothesis. Therefore, it remained uncertain whether the Warburg effect was just a mere by-product of aberrant growth factor signaling until recently, when several studies dealing with genetics demonstrated that the Warburg effect was actually the best fit for tumor growth (but still dispensable under certain conditions as will be developed in Section 2.3) [50,51,52,53,54,55]. Furthermore, as a proof of principle showing the renewed interest in studying tumor cell metabolism during these last years, the number of publications referring to the “Warburg effect” term has increased exponentially since the 2000s [56].

### 2.2. The Warburg Effect and Cellular Growth: A Fine-Tuned Nexus

**Figure 1:**
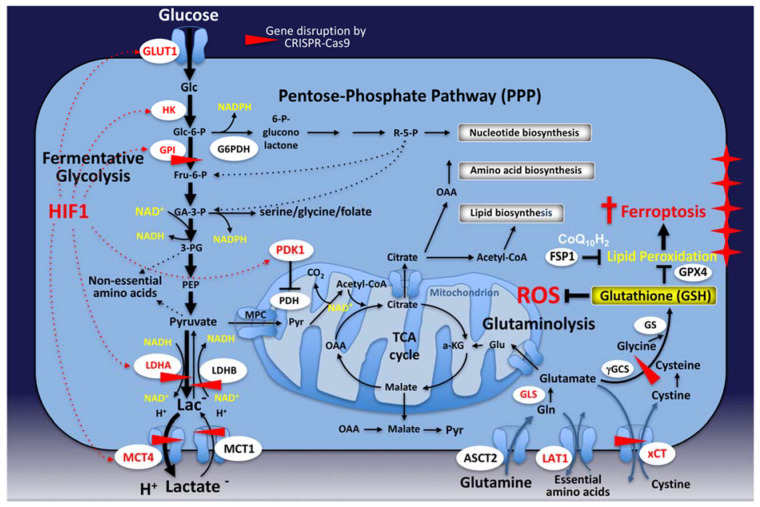
Note from the top—glucose to lactic acid—the three enzymatic steps that have been genetically disrupted in our studies by CRISPR-Cas9 (red horizontal triangles). Red dotted arrows represent the Hypoxia Inducible Factors (HIF)-induced genes that are upregulated during tumor hypoxia including *GLUT1*, *HK*, *GPI*, *LDHA*, *MCT4* and *PDK1*, thereby allowing tumors to have a sustained fermentative glycolysis capacity. Glutathione peroxidase 4 (GPX4) and ferroptosis suppressor protein 1 (FSP1), which is a novel glutathione-independent ferroptosis suppressor, are also depicted in this scheme; both of them prevent the generation of membrane lipid peroxides.

Per unit of glucose, aerobic glycolysis is an unproductive way to favor ATP production in comparison with the amount produced by mitochondrial respiration (2 versus 33.45 molecules of ATP according to the recent observations of Mookerjee et al. [57]), so why would rapidly proliferating cells be dependent on such an unproductive pathway to produce energy? Lactate production from glucose occurs up to 100 times faster than the complete oxidation of glucose that takes place in the mitochondria [58], and simple calculations indicated that the ATP demand may then never reach limiting values in rapidly growing tumor cells [59,60]. Thus, this enhanced glucose consumption is rather used as a source of carbon for anabolic processes and biomass that are needed to favor the growth of rapidly proliferating cells [15,28,29,61,62]. This excess carbon is used for the de novo building-up of nucleotides, lipids, and proteins, and can be redirected into several pathways that arise from glycolysis [15,51,63] (Figure 1). One example is the deviation of the glycolytic flux into the de novo biosynthesis of serine through the phosphoglycerate dehydrogenase (PHGDH) enzyme [59,64]. In addition, it is now admitted that rather than having a rate-limiting demand for ATP, highly dividing cells are in great need of reducing equivalents in the form of NADPH (nicotinamide adenine dinucleotide phosphate) [65,66]. Then, the accrued uptake of glucose allows for a greater synthesis of these reducing equivalents through the oxidative branch of the pentose phosphate pathway (PPP) and the serine synthesis pathway followed by the tetrahydrofolate cycle, which are then used 1) for the maintenance of reduced glutathione (the major cellular antioxidant), and 2) in reductive biosynthesis (most notably in the de novo synthesis of fatty acids) [65,67] (Figure 1). Lastly, the final step of fermentative glycolysis, conducted by lactate dehydrogenase A in reducing pyruvate into lactate with a regeneration of NAD^+^ (nicotinamide adenine dinucleotide), is cardinal by ensuring a glycolytic flux. Lactate serves as a substantial source of energy in rapidly growing tissues/tumors, but also in differentiated organs/tissues subjected to important physiological, nutritional, and energetic demands (such as embryonic and immune cells or regenerating tissues) [51]. For example, during physical exercise, more than half of the energy turnover rate in the heart muscle is recruited from lactate oxidation [68]. In the brain, besides its capacity to support adequate energy levels and the optimal synaptic function [69], lactate per se (and not glucose) was revealed as a key player in alleviating the hypoxia-induced damages of neurons [70]. In rapidly proliferating cancer cells, including lung and pancreatic tumors, lactate could feed the tricarboxylic acid (TCA) cycle; lactate’s contribution as a respiratory fuel exceeded that of glucose, especially in rapidly growing tumors [71,72]. Pyruvate can also be redirected into the mitochondria and converted to acetyl-CoA for entry into the tricarboxylic acid cycle (TCA) [22]. The TCA cycle intermediates are then either oxidized for catabolic purposes or transformed into amino acids or citrate for export back to the cytosol (further details are provided in the following sections). 

**Figure 2:**
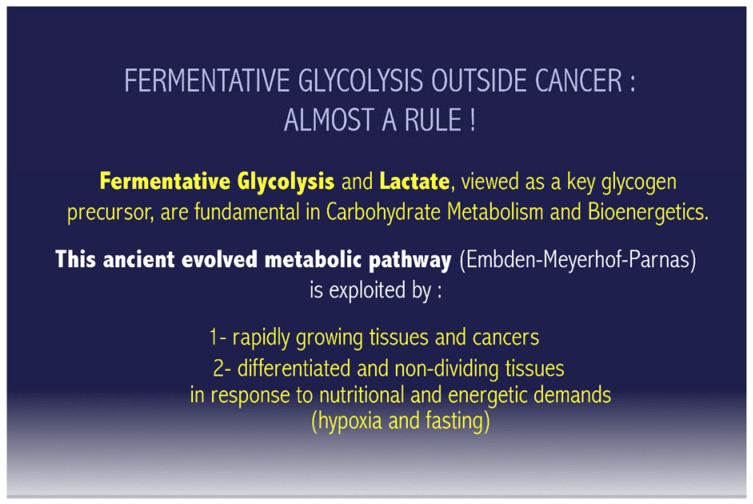
Key conclusions issued from the analysis of fermentative glycolysis (Warburg effect) in normal tissues and cancers [51].

Consequently, this originally thought to be “wasteful” system is ultimately shown to be extremely efficient once one may understand that the ultimate goal of a tumor cell is nothing more than cellular growth and division. Given that the Warburg effect is also noticed during the rapid proliferation of the primary cells, it is more generally perceived as a characteristic of cell proliferation than as a privilege of oncogenic transformation, and can thus be perceived as an important player that contributes to anabolic metabolism (see Figure 2 for a rapid overview) [73,74].

### 2.3. Is the Warburg Effect Dispensable for Cancer?

Cells that sustain high glycolytic rates do not catabolize the totality of their pyruvate into lactate, but rather a significant amount of the pyruvate is oxidized and metabolized in the TCA cycle, thus delivering energy through respiration and the metabolic intermediates for the anabolic pathways that stem from TCA cycle [75]. Hence, as previously reported by Crabtree [39], mitochondrial respiration can actually occur in tumor cells and a key question then arises: is the Warburg effect dispensable for cancer? In other words, would the inhibition of the Warburg effect push tumor cells to rapidly rewire their metabolism towards mitochondria and oxidative phosphorylation (OXPHOS) to survive and proliferate? Our laboratory has even pushed the question further, could full suppression of the Warburg effect arrest tumor growth? We addressed this fundamental question by using two tumorigenic cancer cell lines, derived from human colon adenocarcinoma (LS174T) and mouse B16-F10 melanoma, in which we genetically disrupted the Warburg effect at either one of the three fermentative glycolytic steps: (1) glucose 6 phosphate isomerase, (2) lactate dehydrogenase A, B or both A/B, and (3) lactate/H^+^ symporters monocarboxylate transporter (MCT) 1 and 4 (see Figure 1).

Glucose 6 phosphate isomerase (GPI) is a cytosolic enzyme involved in the reversible inter-conversion between glucose 6 phosphate (G6P) and fructose 6 phosphate (F6P), and its genetic disruption using the CRISPR/Cas9 technique is associated with major metabolic and growth modifications. Indeed, both the LS174T and B16-F10 GPI-KO cell lines, which had no measurable GPI enzymatic activity and secretion of lactic acid, rewired their metabolism towards the PPP and OXPHOS as supported by their augmented respiratory capacity [54]. Remarkably, the highly glycolytic LS174T tumor cell line (with a low respiratory capacity when cultured under standard conditions) displayed a strong reactivation of OXPHOS when challenged by a GPI ablation, thus creating an augmented and exclusive reliance on oxygen [54]. This phenotype explained 1) the inability of the LS174T GPI-KO cells to grow in hypoxia (1% O_2_), and 2) their increased sensitivity to OXPHOS inhibition by phenformin or oligomycin [54]. This vulnerability created by such metabolic rewiring is in line with the observations described for ovarian and hepatocarcinoma cancer cell lines, where GPI silencing completely abolished cancer cell growth in combination with OXPHOS inhibition [76]. 

Lactate dehydrogenase (LDH) is a family of NAD^+^-dependent enzymes that catalyze the reversible conversion between pyruvate and lactate, with a simultaneous oxidation/reduction of the cofactor (NAD^+^/NADH) [77,78]. Active LDH is a homo/heterotetramer assembled from two different subunits: M (muscle) and H (heart), encoded by two separate genes, LDHA (M) and LDHB (H), respectively [77,78]. The important role of LDHA in maintaining a Warburg phenotype and promoting the tumorigenic potential of malignancies was confirmed by numerous findings showing that LDHA inhibition, gene silencing, or knockdown considerably decreased the tumorigenic potential in several types of cancer [50,79,80]. Hence, we used the same genetic approach to create LDHA-KO LS174T and B16-F10 cells and showed that the complete abrogation of the LDHA gene results in only about a 30% decrease in the secreted lactate levels [55]. Interestingly, the LDHA-KO cells revealed an augmented OXPHOS capacity, thus pointing to (1) an increased reliance on OXPHOS for the production of energy, and (2) an amplified vulnerability to respiratory chain inhibitors including phenformin, which significantly reduced the clonal growth [55]. This unexpected low 30% decrease in the secreted lactate levels was corroborated by the finding that the lack of LDHA did not significantly influence in vitro cell growth under normoxic conditions [55]. This could at least partially be explained by the retained ability of LDHA-KO cells to catalyze a pyruvate conversion to lactate, although at a reduced rate compared with the wild type (WT) cells, but at levels sufficient to drive glycolysis, lactate production, and growth. We therefore hypothesized that this activity might be due to the presence of a LDHB isoform in both cell lines, capable of catalyzing the reverse reaction when LDHA is no longer present. However, alone LDHB-KO cells did not significantly impact on the cells’ growth and viability, neither in normoxia nor hypoxia (1% O_2_) [55]. Rather, they behaved essentially like WT cells in terms of lactate secretion, glycolytic and mitochondrial activity, and sensitivity to OXPHOS inhibitors [55]. It was only when both the LDHA and LDHB isoforms were disrupted that a distinct phenotype with no detectable lactate secretion and a complete metabolic redirection toward OXPHOS was observed [55]. It is of importance to note that the genetic LDHA/B-double KO cell phenotype was mimicked in vitro by the dual first specific LDHA, the LDHB inhibitor GNE140 [55], killing the possibility of aberrant emergence during the genetic selection. Again, this increased reliance on OXPHOS displayed by these LDHA/B-double KO cells was marked by an increased vulnerability to the inhibitors that target the mitochondrial respiratory chain (phenformin) in comparison with the WT or LDHA/B-KO cells [55]. Importantly, in vivo, only a delay and not an abrogation of tumor growth could be observed, thus suggesting that at least in immunodeficient mice, the Warburg effect can be replaced by OXPHOS [55]. 

MCTs (1–4) facilitate the transport of lactate and protons into and out of cells and both MCT1 and MCT4 expression levels were evidenced to be increased in tumors [51,81,82,83,84,85]. Having a strong interest in studying these different intracellular pH (pHi)-regulating systems, we investigated the effects of the pharmacological/genetic inhibition of lactic acid export on tumor cells to see whether these cells could sustain metabolic rewiring in order to strive. Pharmacological inhibition with the specific AstraZeneca MCT1/2 inhibitor (AR-C155858) was reported to impede glycolysis and tumor growth of the RAS-transformed fibroblasts expressing only MCT1/2 [86]. Importantly, this compound was shown to be ineffective in preventing the growth of tumor cells lacking glycolytic activity that only rely on OXPHOS to strive [86]. Reciprocally, the expression of MCT4 in respiration-deficient RAS-transformed fibroblasts was shown to favor in vivo tumor growth [83]. In non-small cell lung cancer cells, the genetic disruption of the MCT’s chaperone CD174 was described to lower the glycolytic rate 2.0- to 3.5-fold, and to stimulate mitochondrial respiration, thereby making CD174-null cells particularly sensitive to inhibitors of mitochondrial respiration both in vitro and in vivo [87,88]. Finally, a combined inhibition/disruption of MCT1/4, which severely reduces the lactic acid export, imposes a strong reduction in tumor growth [88,89]. The reason for this growth arrest is due to the intracellular acidification that is known to block mTORC1 [90,91]. Now, this growth arrest/cytostatic effect can be transformed into cell death (energy crisis) when an MCT inhibition is combined with a short exposure to the mitochondrial complex I inhibitor phenformin [88,92,93,94]. 

These three examples point to a common feature: an extraordinary metabolic plasticity displayed by tumor cells. In all three cases, the disruption of the glycolytic flux resulted in an abolishment of the lactic acid secretion and augmented the reliance on OXPHOS for the energetic needs and survival. Coming back to Otto Warburg’s original findings, it has become now evident that the involvement of mitochondrial metabolism to cancer formation and development are of paramount importance. In the following sections, we will specifically focus on mitochondria, by detailing the cancer cell-intrinsic/extrinsic mechanisms through which they can impact on malignant transformation and progression, and briefly emphasize the therapeutic potential of targeting mitochondrial metabolism in cancer therapy.

## 3. Mitochondrial Metabolism in Cancer

### 3.1. Carcinogenesis

Mitochondria whose name was derived from the Greek “mito”, meaning thread, and “chondria”, due to their granulosity [95], may favor a malignant transformation by at least two major mechanisms that include (1) the mitochondrial reactive oxygen species (ROS) that support the accumulation of oncogenic DNA alterations and activation of oncogenic pathways [96], and (2) the abnormal increase in the specific mitochondrial metabolites including fumarate, succinate, and 2-hydroxyglutarate (2-HG), which has important transforming properties [97].

#### 3.1.1. Reactive Oxygen Species (ROS)

ROS, in the form of superoxide anion radical and/or hydroxyl radical are produced from physiological metabolic reactions and were described for the first time by Poul K. Jensen in 1966, who proved that they originate from the respiratory chain [98,99]. Later on, Chance and co-workers also reported that isolated mitochondria produce H_2_O_2_ [100,101,102]. Indeed, through the mitochondrial complexes I, II and III, OXPHOS has been identified as the main source of ROS production, thus making mitochondria the major contributors of cellular redox homeostasis [98,103]. Their participation during a malignant transformation is shown by the fact that mice deficient in p53 that are maintained under hypoxic conditions (10% O_2_) display a reduced level of tumorigenesis, and thus a survival advantage in comparison with those exposed to the standard atmospheric conditions (21% O_2_) [104]. In line with this, the autophagic removal of damaged mitochondria overproducing ROS led to a reduced potential of oncogenesis, since the specific knockdown or knockout of autophagic genes (such as *Atg5* or *Atg7*) have been shown to promote cancer transformation [105,106,107]. ROS are also involved in oncogenic signal transduction cascades via cysteine oxidation, as demonstrated with H_2_O_2_, which can inactivate the tumor suppressor phosphatase and tensin homolog (PTEN) by oxidizing the active site’s cysteine residues [103,108]; this leads to the formation of a disulfide bond and prevents PTEN from inactivating the phosphoinositide 3 kinase (PI3K) pathway [96,109]. ROS may have several yet to be discovered consequences on the diverse mitogen-activated pathways that are usually abrogated by phosphatases [109,110]. Kamata et al. indeed demonstrated that the accumulation of intracellular H_2_O_2_ could inactivate mitogen-activated protein kinase (MAPK) phosphatases through oxidation of their catalytic cysteine, and thus maintains the MAPK pathway in an active state [111,112]. In line with this, RAS and RAC small GTP-binding proteins appeared to be directly linked to the production of superoxide anion radical O_2_^−^ in transformed fibroblasts; an ROS-mediated neo-transformation of these cells could then be evidenced since treatment with antioxidants was associated with a block in the RAS-induced cellular transformation [113]. Further, the accumulation of ROS can directly affect DNA integrity and it has been shown that ROS-mediated DNA damages could favor the initiation stage of tumorigenesis. For example, the capacity of hydroxyl radicals to attack DNA is well known and was shown to trigger single and/or double strand breaks which can then affect genome integrity [114]. In addition to causing genetic modifications, ROS have also been associated with epigenetic alterations that favor oncogenic transformation; indeed, a ROS-induced hypermethylation of the promoter region of tumor suppressor genes has been shown to promote carcinogenesis, as exemplified in liver cancer, where hepatocellular carcinoma (HCC) cells exposed to H_2_O_2_ had increased hypermethylation levels of the promoter region of the E-cadherin gene (a hallmark of an epithelial-to-mesenchymal transition (EMT) that is lost during this process), leading then to its down-regulation [115].

#### 3.1.2. Oncometabolites

Dominant mutations in mitochondrial enzymes allowed the discovery of mitochondrial-derived signaling molecules that are called oncometabolites. Succinate dehydrogenase complex iron sulfur subunit B (SDHB), fumarate hydratase (FH), and the cytosolic and mitochondrial isocitrate dehydrogenase (IDH) isoforms 1 and 2 have been shown to be mutated in various types of cancer [97]. While SDHB and FH enzymes are generally affected by the loss-of-function mutations, with augmented levels of fumarate and/or succinate, IDH1 and IDH2 often display gain-of-function mutations that lead to the production of 2-HG [116]. Behaving as bona fide oncometabolites, the accumulation of fumarate, succinate, and 2-HG may be enough to enhance tumor transformation [116]. The structural similarity of these oncometabolites to α-ketoglutarate (α-KG) makes them of interest as they can act as competitive inhibitors of the α-KG-dependent enzymes that regulate gene expression levels through epigenetic modifications, including the Jumonji domain (JMJ) histone lysine demethylases and ten-eleven translocation (TET) dioxygenases [117,118]. For example, TET activity abrogation was reported to increase the hypermethylation of CpG islands, leading then to the silencing of genes [117]. 2-HG and succinate were shown to alter the α-KG-dependent HIF-prolyl oxidase activity of the egl-9/PHD family hypoxia inducible factor 1α and 2 (EGLN1/PHD2—EGLN2/PHD1), hence favoring neo-transformation through a mechanism associated with the stability of the hypoxia inducible factors 1 and 2 [119,120,121]. Fumarate can also promote a non-enzymatic post-translational protein modification known as “succination”, most likely due to the inactivation of the succinate dehydrogenase enzyme complex, and augments the kelch-like ECH-associated protein 1 (KEAP1), enabling the activation of the transcription factor’s nuclear factor erythroid 2-related factor 2 (NRF2) and consequent upregulation of antioxidant pathways [122]. The same post-translational modification also seems to affect the non-enzymatic antioxidant glutathione, thereby preventing its recognition by glutathione reductase and resulting in decreased NADPH and an augmented ROS production [123]. Altogether, these observations perfectly represent the critical influence that mitochondria can exert at the different stages of malignant transformation.

### 3.2. Cancer Progression

#### 3.2.1. A Biosynthetic Hub

As progressing tumors can rely on the reversibility of various TCA cycle reactions and the existence of multiple anaplerotic circuitries centered on mitochondria to survive, the following paragraphs will discuss the different TCA cycle intermediates that are involved in this phenomenon to see how mitochondria represent a powerhouse from which anabolism is fed [124,125]. (see Figure 1 for a general and non-exhaustive overview)

Located at a central position, citrate is a key intermediate that operates as a major node of flexibility [126] (Figure 1). Besides replenishing the oxidative mode of the TCA, citrate can also be transformed into acetyl-CoA for export to the cytosol and nucleus, where it can be used as a substrate either for the synthesis of fatty acids and cholesterol, or for acetylation reactions [127]. Indeed, upregulation of lipogenesis was proposed as a characteristic of rapidly growing tumors to support the membrane need associated with an intense proliferation [128]. The inhibition of ATP-citrate lyase (ACLY), which catalyzes mitochondrial-derived citrate into acetyl-CoA, has been shown to reduce tumorigenesis in several models [128]. In contrast, certain cancers rely on mitochondrial fatty acid oxidation (FAO) for ATP generation [129]. Further, FAO may be a favored fuel choice for cancers undergoing stress as evidenced in HCC, where highly tumorigenic HCC cells increased FAO to favor ATP generation when cultured under glucose-restricted conditions [28,29]. Beyond ATP production, increased FAO in some cases may also confer benefits in maintaining redox homeostasis [129]. It was also reported that an epigenetic remodeling of chromatin could result from the production of FAO-derived acetyl-CoA, subsequently causing gene expression modifications, leading ultimately to enhanced cell proliferation [130]. In line with this, an ACLY-dependent production of acetyl-CoA from mitochondrial-derived citrate is also used for epigenetic modifications: (1) with the modification of histone acetylation patterns that ultimately leads to chromatin modification via histone acetyl transferases (HATs), and (2) for the acetylation of many cytosolic and mitochondrial proteins, thereby modifying their structural conformation and activity [127,131]. 

Glutamine can be oxidized through the TCA cycle and represents an important source for the synthesis of macromolecules [75] (Figure 1). The amide nitrogen on glutamine is used in purine synthesis, whereas glutamine-derived carbons are used in pyrimidine, amino acid and lipid synthesis. Glutaminolysis, which corresponds to glutamine catabolism, was reported to be (1) augmented in numerous tumors that are addicted to glutamine, and (2) often induced by MYC upregulation of glutaminase (GLS), which catabolizes glutamine into glutamate and ammonia [75,132]. Glutamate is then oxidized to α-KG by glutamate dehydrogenase (GDH), thus allowing the entry into the TCA cycle. For example, the expression of Sirtuin 4 (SIRT4) in B cell lymphoma, which is a mitochondrial-localized tumor suppressor protein that inhibits a glutamate-to-α-KG conversion, was associated with a decreased glutamine uptake and cellular growth; conversely, its loss increased glutamine consumption and hastened tumorigenesis [133]. In parallel, it was indicated that, in comparison to quiescent cells, proliferating cells (normal and tumor) primarily use glutamate via transaminases to couple non-essential amino acid synthesis to α-KG production and TCA cycle anaplerosis, pinpointing the relevance of this pathway in supporting biosynthesis during tumor cell proliferation [134]. Glutamine also acts as another source of carbons for acetyl-CoA production, which can then serve lipid biosynthesis through reductive carboxylation under hypoxia or when the mitochondria are damaged [135,136]. Experiments using a [5-^13^C]-glutamine tracer could indeed demonstrate that, through reductive carboxylation, an up to 25% formation of lipogenic acetyl-CoA came from glutamine in several types of tumor cells [135]. In addition, via malic enzyme activity, glutamine-derived malate is also metabolized into pyruvate, which can then be converted into oxaloacetate/acetyl-CoA for reentry into the TCA cycle [137]. In line with this, cytosolic malic enzyme 1 was demonstrated to sustain NADPH generation from glutamate in pancreatic and breast cancers, thereby participating in redox homeostasis [134,138]. Furthermore, NADPH production can also be achieved via the IDH1-dependent reductive carboxylation of glutamine, thereby reducing the levels of mitochondrial ROS [136] (for further details on glutamine metabolism and redox homeostasis, refer to [136,139,140]). Importantly, while several studies could provide strong evidence that the requirements for glutamine were substantial for numerous types of tumors [141], it is important to note that the dispensability of glutamine was also noted under certain circumstances, as evidenced by lung tumor cells, which lost their glutamine dependency when grown in vivo [142].

Mitochondria also participate to malignant progression through nucleotide synthesis via one-carbon metabolism [143]. The mitochondrial folate synthesis pathway consists of serine hydroxylmethyltransferase (SHMT2) and the bifunctional methylenetetrahydrofolate dehydrogenase/cyclohydrolase (MTHFD2) [144,145]. Gene expression profiling could indeed identify many types of MTHFD2-overexpressing tumors and further studies revealed MTHFD2 as an important determinant of cancer cell survival [145]. Further, SHMT2 expression was detected in ischaemic tumor regions, thereby procuring a proliferative advantage under hypoxia [146]. Using isotope labeling, Kim et al. demonstrated that while inhibition of SHMT2 is associated with an improved pyruvate kinase (PKM2) activity and carbon flux into the TCA cycle, cells that display greater levels of SHMT2 activity limit that of PKM2 and flux into the TCA. This action decreases oxygen consumption and confers to cells localized in weakly vascularized tumor zones a survival benefit [146]. Additionally, SHMT2 regulation of serine metabolism is also involved in the generation of NADPH, and thus the subsequent detoxification of ROS under hypoxia, which is an essential function for survival of MYC-driven cancers [144]. Altogether, these observations emphasize the crucial role of mitochondria in allowing cancer cells to thrive during malignant progression. 

#### 3.2.2. Resistance to RCD

As aforementioned, progressing tumors encounter fluctuating microenvironmental conditions and this would commonly create an accrued sensitivity to mitochondrial regulated cell death (RCD) via a mitochondrial outer membrane permeabilization (MOMP) or a mitochondrial permeability transition (MPT) [147]. The pro-apoptotic BCL-2 family members BAX (BCL-2-associated X protein) and BAK (BCL-2 homologous antagonist killer) are recruited to the mitochondrial outer membrane and oligomerize to mediate MOMP, resulting in a pore formation and cytochrome c release from mitochondria into the cytosol to initiate caspases activation [148]. While BAX/BAK is inhibited by anti-apoptotic proteins under physiological conditions, neoplastic cells evade this via the downregulation of pro-apoptotic and/or upregulation of anti-apoptotic genes by several mechanisms (for further details see [149]). Therefore, the balance of pro- and anti-apoptotic proteins impacts a tumor cell’s vulnerability in response to apoptotic signals. Additionally, since some tumors sustain an increased mitochondrial transmembrane potential (ΔΨm) associated with high glycolytic rates and increased resistance to RCD, restoring pyruvate consumption via a chemical inhibition of PDK1 was not only reported to augment RCD sensitivity, but also inhibit in vivo tumor growth [150]. In line with this, detaching mitochondrial-bound hexokinase II (enzyme involved in the first step of glycolysis by converting glucose into G6P) from mitochondria has been suggested to trigger MOMP in different types of cancer cells [151]. The maintenance of optimal antioxidant defenses is also crucial for tumor cells to evade ROS-driven MPT as evidenced by Vaughn et al., who demonstrated that both normal/tumor cells thoroughly impede cytochrome c-mediated apoptosis via a mechanism that relies on glucose metabolism; cytochrome c is indeed reduced and held inactive by the PPP-derived NADPH [152]. Mitochondrial dynamics also dictate apoptotic susceptibility, as the loss of dynamin-related protein 1 (DRP1) (a fundamental component of mitochondrial fission) was shown to delay cytochrome c release and apoptotic induction [153]; similarly, a decreased DRP1 expression was shown to enhance resistance to an oncogenic V-Ki-ras2 Kirsten rat sarcoma viral oncogene homolog (KRAS)-induced cellular transformation [154]. The relevance of mitochondrial dynamics in apoptosis is further evidenced by the induction of mitochondrial hyperfragmentation secondary to mitofusin 1 (MFN1) ablation—an essential component of mitochondrial fusion—resulting in an augmented resistance to apoptotic stimuli owing to the loss of the interaction between BAX and the mitochondrial membranes [155]. Moreover, the inhibition of DRP1 was shown to restore sensitivity to apoptotic stimuli through the reestablishment of a balanced and adequate mitochondrial network [155].

#### 3.2.3. Metastatic Dissemination

Metastases generally refers to the dissemination of tumor cells to distant organ sites [156]. One of the initial changes of the metastatic cascade is EMT induced under hypoxia, which bestows tumor cells with augmented invasive properties [157,158,159]. Multiple mitochondrial metabolites have been shown to support the EMT process, and especially fumarate, which could counteract the transcription of anti-metastatic microRNAs upon the inhibition of TET dioxygenases [160,161,162]. Mitochondrial biogenesis and OXPHOS have also been suggested to support metastatic dissemination, as demonstrated when the peroxisome proliferator-activated receptor gamma coactivator-1 alpha (PGC-1α) was silenced in breast cancer models [163]; PGC-1α is a central regulator of mitochondrial biogenesis whose levels are often associated with tumor reliance on mitochondrial respiration (for further details refer to [164]). While circulating cancer cells originating from orthotopic mammary tumors displayed greater mitochondrial biogenesis and respiration, the silencing of PGC-1α resulted in a reduced migratory phenotype and metastatic potential [163]. Moreover, rotenone, which is a mitochondrial complex I inhibitor, mirrored the PGC-1α silencing effects on tumor cells, with reduced mitochondria respiration and invasive properties, thereby pinpointing the crucial role of mitochondrial-derived ATP on the capacity of tumor cells to be invasive; these results brought newfound significance to the role of PGC-1α in supporting tumor metastasis [163]. ROS were also evidenced to activate multiple signal transduction cascades associated with increased metastatic capabilities, such as proto-oncogene tyrosine-protein kinase SRC and protein tyrosine kinase 2 beta (PTK2B) signaling [165,166]. Indeed, the inhibition of SRC was evidenced to impede tumor cell migration and mitochondrial ROS scavenging to block metastatic take in rodents [165]. Imbalances in the mitochondrial shape also dictate the metastatic susceptibility as a consequence of mild ROS overproduction. Indeed, an augmented expression of DRP1 was associated with a migratory phenotype in several types of cancer cells [167]. In contrast, under severe oxidative stress conditions, ROS have been shown to abrogate metastasis mainly as a straight consequence of weakened fitness, RCD, and/or cellular senescence [168,169]. OXPHOS and subsequent ROS generation are therefore required for the metastatic cascade, and collectively these observations highlight the fundamental role held by mitochondria in supporting cancer progression and metastasis. 

As discussed in the above sections, the diversity of the carbon substrates fueling tumor cells thus perfectly exemplifies the concept of metabolic heterogeneity, which is also and importantly mirrored by the different components found in the tumor microenvironment. To take the reasoning further, carcinoma-associated fibroblasts (CAFs) have indeed been evidenced to dynamically participate in the metabolic needs of tumor cells, thereby favoring tumor initiation and progression [170,171,172]. Of note, an elegant work from Mechta-Grigoriou’s group recently identified two particular subsets of CAFs having prominent roles in inducing tumor cell migration and invasion in breast cancer [173]. 

## 4. Therapeutic Challenges

The final objective of traditional chemotherapeutics and anti-cancer agents is to prompt the death of tumor cells [174]. Since mitochondria play a crucial role in the control of regulated cell death (RCD), emerging evidence has suggested that targeting mitochondrial metabolism could potentially be a promising area for cancer treatment. As previously stated, tumor cells can interchangeably use glycolysis and OXPHOS as a source of ATP production (see Section 2.2 and Section 2.3). Hence, although targeting mitochondrial ATP generation might not be an appealing strategy for cancer treatment, we however, propose that there are at least three reasons to envision it as a potential target since (1) the interiors of various solid tumors remain weakly perfused and the ETC is still functioning; a drug preventing mitochondrial ATP generation would then favor the cell death of these weakly oxygenated tumors [175,176], (2) there are tumors that heavily rely on OXPHOS to generate ATP, and that these cancer cells are likely to be imaged by ^18^F-BnTP, a recently reported promising imaging tool sensitive to drugs that restrict mitochondrial ATP production due to a lack of glycolytic compensation [86,88,177,178,179]; important to mention as well is IACS-010759, a selective inhibitor of mitochondrial complex I [180], and (3) preventing mitochondrial ATP generation would synergize with therapeutic strategies that reduce glycolysis. However, one of the main challenging issues with targeting mitochondria is to not impact on normal cells; the prerequisite here, would be whether tumors preferentially uptake the drugs that prevent the production of mitochondrial ATP in comparison with normal cells. In this regard, we speculate that a metformin/phenformin drug may be a promising anti-cancer agent that targets mitochondrial ATP generation without increasing the toxicity in normal tissues. Similarly, it has also been proposed that decreasing the mitochondrial protein translation and stability may offer another opportunity to obstruct mitochondrial bioenergetics. Indeed, tetracycline treatment was shown to impede mitochondrial protein translation and potentiate anti-tumor effects in several preclinical models of leukemia; dismantling the translation of ETC-related proteins resulted in major defects in mitochondrial respiration [181]. As said, glutamine is an important carbon source for cancer cells. Targeting glutamine metabolism and transport becomes of great interest [27], as evidenced with two specific GLS inhibitors: compound 968 and bis-2-(5-phenylacetamido-1,2,4-thiadiazol-2-yl)ethyl sulfide, which reduced glutamine catabolism and delayed in vivo tumor growth in experimental models of cancer [137,182]. Similarly, inhibiting the glutamate-to-α-KG conversion by aminotransferases also decreased tumor growth [183,184]. Notably, one may keep in mind that not all malignancies display an addiction to glutamine catabolism, and using [^13^C]-glutamine infusion in patients may help in predicting which tumors will respond to therapies that target glutamine metabolism [185]. As stated above, mitochondria and the signaling pathways that are responsive to ROS are intrinsically linked and targeting mitochondrial ROS through antioxidants has shown to efficiently hamper tumorigenesis both in vitro and in vivo [22]. However, many clinical studies showed that the effects of antioxidant treatment on the onset and progression of tumors are extraordinarily difficult to anticipate. It has indeed been reported that moderate/high increases of ROS could unexpectedly act as cancer suppressors by maintaining senescence-induced tumor suppression and sensitizing tumor cells to chemotherapeutic drugs, thus making the use of antioxidant treatments very limited and most of the time ineffective [22,186]. On the other side, to counterbalance the enhanced ROS production, tumor cells require NADPH and one way to achieve this is using one-carbon metabolism [22]. In this regard, beyond the critical identified role of SHMT2 in regulating redox homeostasis (via NADPH production) [144], MTHFD2 was also reported to be essential since its loss resulted in an improved capacity of sensitizing tumor cells to oxidant-induced cell death [145]. Targeting mitochondrial metabolic enzymes in combination with other therapies known to augment the production of ROS should then be considered and may eventually offer new promising therapeutic avenues. 

In line with these last observations, ferroptosis has recently been described as an iron-dependent form of non-apoptotic cell death that can be activated in cancer cells by natural stimuli, synthetic agents, or the disruption of the cystine/glutamate antiporter xCT [66,187] (Figure 1). Three key hallmarks define ferroptosis, namely the oxidation of polyunsaturated fatty acid (PUFA)-containing phospholipids, the availability of redox-active iron and the loss of the glutathione peroxidase 4 (GPX4) [188,189,190] (Figure 1). Although it is uncertain whether mitochondria could have a function in this process, recent evidence could bring new findings on novel functions that impact on ferroptosis such as the status of ferroptosis suppressor protein 1 (FSP1) in the control of the reduced co-enzyme Q_10_ [191], which may potentially highlight the role of ferroptosis as a novel attractive therapeutic concept in cancer biology [192,193]. However, since no consensus on the role of mitochondria during ferroptosis has been reached, further investigations are still required.

Finally, altogether, these observations exemplify the intricate contribution of mitochondria during malignant transformation and progression, and how targeting tumor mitochondrial metabolism actually remains a big challenge. 

## 5. Conclusions

In contrast to what Otto Warburg stated almost a century ago, the respiration of all cancer cells is not damaged and even more, it has become evident that mitochondrial metabolism does influence the functionality and aggressiveness of tumor cells. Mitochondria are indeed multifaceted organelles that impact on malignant initiation, growth, and survival, and multiple aspects of mitochondrial biology beyond ATP generation dynamically support the onset and development of tumors. Furthermore, the substantial flexibility that mitochondria confer to neoplastic cells, such as modifications in fuel choice utilization, bioenergetics, oxidative stress, and susceptibility to cell death, allows the survival of these cells in the face of hostile fluctuating microenvironmental conditions. The rapid metabolic shift of tumor cells towards OXPHOS when the Warburg effect is abrogated perfectly exemplifies this mitochondrial-induced flexibility and remarkably highlights the existence of a metabolic/bioenergetic continuum shared between glycolysis and mitochondria (OXPHOS). Importantly, tumor cell metabolism should not be perceived as a binary concept in which tumor cells only rely on the Warburg effect on one side or on OXPHOS on the other, but rather to an integrative system that displays different metabolic phenotypes and adaptations according to the location within the tumor microenvironment. Indeed, new evidence could reveal a dual capacity of tumor cells in sustaining both glycolysis and OXPHOS [172]. Finally, mitochondrial metabolism has brought significant attention as a target for the expansion of innovative anti-cancer agents. However, significant issues in translating these preclinical drugs (that were evidenced to be highly effective in eradicating tumorigenic cancer cells) towards clinical settings still remain, as their use would necessarily affect normal cells, including beneficial anti-cancer immune cells. Therefore, sophisticated therapeutic strategies in order to precisely modulate mitochondrial functions in a distinct cellular type will have to be defined in the future.

## Abbreviations

2-HG	2-Hydroxyglutarate
ACLY	ATP-citrate lyase
CAFs	Carcinoma-associated fibroblasts
DRP1	Dynamin-related protein 1
EMT	Epithelial-to-mesenchymal transition
F6P	Fructose-6-phosphate
FAO	Fatty acid oxidation
FDG-PET	Fluorodeoxyglucose-positron emission tomography
FH	Fumarate hydratase
FSP1	Ferroptosis Suppressor Protein 1
G6P	Glucose-6-phosphate
GDH	Glutamate dehydrogenase
GLS	Glutaminase
GPI	Glucose-6-phosphate isomerase
GPX4	Glutathione peroxidase 4
HATs	Histone acetyl transferases
HCC	Hepatocellular carcinoma
HIF	Hypoxia Inducible Factors
IDH	Isocitrate dehydrogenase
JMJ	Jumonji domain
KEAP1	Kelch like ECH-associated protein 1
KRAS	V-Ki-ras2 Kirsten rat sarcoma viral oncogene homolog
LDH	Lactate dehydrogenase
MAPK	Mitogen-activated protein kinase
MCT	Monocarboxylate transporter
MFN1	Mitofusin 1
MOMP	Mitochondrial outer membrane permeabilization
MPT	Mitochondrial permeability transition
MTHFD2	Methylenetetrahydrofolate dehydrogenase/cyclohydrolase
NAD^+^	Nicotinamide adenine dinucleotide
NADPH	Nicotinamide adenine dinucleotide phosphate
NRF2	Nuclear factor erythroid 2-related factor 2
OXPHOS	Oxidative phosphorylation
PGC-1α	Peroxisome proliferator-activated receptor gamma coactivator-1 alpha
PHGDH	Phosphoglycerate dehydrogenase
pHi	Intracellular pH
PI3K	Phosphoinositide 3 kinase
PKM2	Pyruvate kinase
PPP	Pentose phosphate pathway
PTEN	Phosphatase and tensin homolog
PTK2B	Protein tyrosine kinase 2 beta
PUFA	Polyunsaturated fatty acid
RCD	Regulated cell death
ROS	Reactive oxygen species
SDH	Succinate dehydrogenase complex iron sulfur subunit
SHMT2	Serine hydroxylmethyltransferase
SIRT4	Sirtuin 4
TCA cycle	Tricarboxylic acid cycle
TET dioxygenases	Ten-eleven translocation dioxygenases
WT	Wild Type
α-KG	α-Ketoglutarate
